# Editorial—Special Issue on Hypertrophic Cardiomyopathy

**DOI:** 10.3390/biomedicines14020325

**Published:** 2026-01-30

**Authors:** Celestino Sardu

**Affiliations:** 1 Department of Advanced Medical and Surgical Sciences, University of Campania “Luigi Vanvitelli”, 80126 Naples, Italy; drsarducele@gmail.com; Tel.: +39-0815665110; Fax: +39-0815095303; 2 Pollution and Cardiovascular Diseases Research Centre, University of Campania “Luigi Vanvitelli”, 80126 Naples, Italy; 3 Department of Cardiovascular Sciences, Responsible Research Hospital, 86100 Campobasso, Italy; 4 Department of Neuro-Cardiological Sciences, IRCCS Neuromed, 86077 Pozzilli, Italy

Hypertrophic cardiomyopathy (HCM) is one of the most complex and heterogeneous conditions in cardiovascular medicine [1]. HCM is a lifelong, dynamic disorder with broad phenotypic expression and variable clinical trajectories, its therapeutic approaches are consistently evolving [1]. A more in-depth understanding of HCM pathophysiology has facilitated genetic characterization and clarified the central role of sarcomeric mutations [1]. This has highlighted the influence of modifier genes, epigenetic mechanisms, and environmental factors on disease expression. Additionally, improvements in imaging techniques, including advanced echocardiography and cardiac magnetic resonance, have enhanced phenotypic stratification, enabling earlier diagnosis, more precise risk assessment, and a refined evaluation of myocardial remodeling, fibrosis, and microvascular dysfunction [2].

Despite these advances, significant knowledge gaps persist. However, the risk stratification for sudden cardiac death, particularly in borderline- or intermediate-risk patients, is an open debate [1,2]. The mechanisms linking genotype to phenotype are not fully understood, and the determinants of disease progression, such as from asymptomatic hypertrophy to heart failure, atrial fibrillation, or end-stage disease, are still being elucidated [1,2]. In this context, therapeutic approaches have historically focused on symptom relief rather than disease modification, underscoring the need for therapies that directly target underlying molecular abnormalities [3]. This current Special Issue, titled “Advanced Research in Hypertrophic Cardiomyopathy,” was conceived to address these unmet needs by bringing together contemporary research spanning basic science, translational investigation, and clinical practice. The contributions collectively reflect the multidimensional nature of HCM, emphasizing not only traditional aspects such as diagnosis and risk assessment, but also emerging concepts related to myocardial energetics, inflammation, arrhythmogenesis, and cardiometabolic interactions [4]. Importantly, the Special Issue highlights the transition of HCM research toward precision medicine, integrating genetic, imaging, and clinical data to inform individualized patient care.

Several priorities should guide future HCM research toward a multi-step diagnostic and therapeutic approach. First, there is a critical need for longitudinal, genotype-informed studies that clarify disease trajectories and identify early markers of progression. Second, the development and validation of novel disease-modifying therapies, particularly those targeting sarcomeric function and downstream signaling pathways, could represent a paradigm shift and require robust clinical evidence across diverse patient populations. Third, greater attention should be paid to comorbidities, aging, and sex-related differences, which increasingly shape outcomes in real-world HCM cohorts. Finally, implementation of scientific research will be essential to make sure that advances in diagnostics and therapeutics translate into equitable, guideline-concordant care across healthcare systems, ensuring that HCM research is able to enter into a transformative era. Furthermore, by addressing key knowledge gaps and highlighting innovative approaches, this Special Issue aims to stimulate further investigation and interdisciplinary collaboration. Continued progress will depend on integrating mechanistic insight, clinical rigor, and patient-centered perspectives, ultimately improving outcomes for individuals living with hypertrophic cardiomyopathy.
